# The Role of DICOM in Artificial Intelligence for Skin Disease

**DOI:** 10.3389/fmed.2020.619787

**Published:** 2021-02-10

**Authors:** Liam J. Caffery, Veronica Rotemberg, Jochen Weber, H. Peter Soyer, Josep Malvehy, David Clunie

**Affiliations:** ^1^Centre for Online, Centre for Health Services Research, The University of Queensland, Brisbane, QLD, Australia; ^2^Dermatology Research Centre, The University of Queensland Diamantina Institute, The University of Queensland, Brisbane, QLD, Australia; ^3^Dermatology Service, Department of Medicine, Memorial Sloan Kettering Cancer Center, New York, NY, United States; ^4^Department of Dermatology, Princess Alexandra Hospital, Brisbane, QLD, Australia; ^5^Department of Dermatology, Institut d'Investigacions Biomèdiques August Pi i Sunyer, Hospital Clinic of Barcelona, University of Barcelona, Barcelona, Spain; ^6^PixelMed Publishing, Bangor, PA, United States

**Keywords:** dermatology, artificial intelligence, DICOM, standards, imaging

## Abstract

There is optimism that artificial intelligence (AI) will result in positive clinical outcomes, which is driving research and investment in the use of AI for skin disease. At present, AI for skin disease is embedded in research and development and not practiced widely in clinical dermatology. Clinical dermatology is also undergoing a technological transformation in terms of the development and adoption of standards that optimizes the quality use of imaging. Digital Imaging and Communications in Medicine (DICOM) is the international standard for medical imaging. DICOM is a continually evolving standard. There is considerable effort being invested in developing dermatology-specific extensions to the DICOM standard. The ability to encode relevant metadata and afford interoperability with the digital health ecosystem (e.g., image repositories, electronic medical records) has driven the initial impetus in the adoption of DICOM for dermatology. DICOM has a dedicated working group whose role is to develop a mechanism to support AI workflows and encode AI artifacts. DICOM can improve AI workflows by encoding derived objects (e.g., secondary images, visual explainability maps, AI algorithm output) and the efficient curation of multi-institutional datasets for machine learning training, testing, and validation. This can be achieved using DICOM mechanisms such as standardized image formats and metadata, metadata-based image retrieval, and de-identification protocols. DICOM can address several important technological and workflow challenges for the implementation of AI. However, many other technological, ethical, regulatory, medicolegal, and workforce barriers will need to be addressed before DICOM and AI can be used effectively in dermatology.

## Introduction

The Digital Imaging and Communications in Medicine (DICOM) standard (1) is a comprehensive, international standard for medical imaging. DICOM defines a standard medical image file format and a metadata model and network services for the storage, transmission, and query and retrieval of objects. DICOM has also specified other network services to improve the efficiency of medical imaging workflows.

The most common DICOM data object is an image file. A DICOM image file consists of metadata and the pixel data of the image melded into a single file. The pixel data may be encoded using the Joint Photographic Expert Group format or other standard compression methods. The metadata model is standardized by object type, meaning there are different metadata models for, say, a magnetic resonance image, and a photographic image. The metadata model is described in an Information Object Definition (IOD). An IOD consists of an amalgamation of modules, where each module is a collection of patient, study, equipment, and image metadata attributes.

Although DICOM is ubiquitous in some medical image-producing specialties (e.g., radiology, cardiology, and radiation therapy), to date, the use of DICOM for dermatological imaging has been limited. There is, however, a growing realization that the adoption of DICOM for dermatological imaging is advantageous compared with other methods of image management (2). Clinical imaging for dermatology can be encoded using the existing, visible light photography IOD. Dermatology-specific IODs for dermoscopy have been developed and included in the DICOM standard. Future work items proposal will be for total body photography and reflectance confocal microscopy (3).

One of the primary motivations for adopting DICOM for dermatology imaging has been interoperability with digital health ecosystems such as electronic medical records, picture archiving and communication systems (PACS), and enterprise imaging repositories. In addition to these interoperability benefits, DICOM adoption is also likely to facilitate the use of artificial intelligence (AI) in clinical dermatology.

The application of AI for dermatology most often uses an image as input into a machine learning algorithm and results in the algorithm outputting a diagnostic or risk prediction of a disease condition. This use of AI is a form of image classification, and convolutional neural networks (CNNs) are becoming the most promising of AI algorithms for dermatology image classification (4). Melanoma diagnosis is a common application of dermatology image classifiers for which several reader studies have found that under experimental conditions, the diagnostic performance of AI can match that of an experienced dermatologist (5–7). Similar results have been reported for psoriasis (8) and onychomycosis (9).

DICOM is an evolving medical imaging standard with continual additions to the standard in the form of supplements and corrections. The increasing interest in AI image classifiers in medical imaging is, in turn, catalyzing the inclusion of AI-specific content in the DICOM standard. The DICOM Standards Committee has a dedicated AI working group (WG 23). The role of this working group is to develop a DICOM mechanism to support AI workflows and encode AI artifacts (10). This paper aims to discuss the role of DICOM in the AI in the context of dermatological imaging and review the current status of AI-specific content in the DICOM standard.

## Derived Objects

Preprocessing and post-processing of images for prediction analysis by machine learning create derived image objects such as resized or down-sampled images, segmentation images, visual explanation (e.g., saliency maps) images, and the algorithm's output. Research has shown that the output of an AI model influences the clinical decision of even experienced dermatologists (7, 11). Hence, derived objects should form part of the patient's medical record if AI is used in clinical practice to meet the health-care provider's regulatory and legal obligation to retain medical records. In addition to regulatory and legal requirements to store derived objects, there are also ethical aspects. The Joint European and North American Multi-society Statement on the Ethics of AI in radiology (12) recommended that the output of AI algorithms need to be stored in an auditable format to permit the investigation of errors and the monitoring over time to ensure there is no degradation of performance.

In practical terms, encoding the derived objects as DICOM objects with the appropriate identifying and descriptive metadata would allow them to be stored alongside the original image files in the health-care organization PACS or enterprise imaging repositories and visualized in conjunction with the clinical images.

Further, the use of DICOM is likely to improve image acquisition and image review workflows. AI has been shown to substantially reduce the amount of time radiologists spend on image review without compromising diagnostic accuracy (13–15). The same potential exists in dermatology, and the greatest efficiency gains are likely to be achieved for dermatologists reviewing total skin imaging studies. In hospitals, medical imaging infrastructure, including acquisition devices and image repositories, nearly all use DICOM as the underlying communication protocol. Speaking a common language can improve the efficiency of workflows, for example, automatic population of patient demographics at acquisition or the automated storage of images to an image repository. Implementing AI as a standalone product, as opposed to being integrated within the medical imaging ecosystems, may negate the diagnostic efficiencies afforded by using AI.

Original images often need to be resized to a fixed image size before inputting into a CNN. In some instances, we have seen dermoscopic images resized to a fixed resolution of 224 × 224 pixels (7). However, other datasets have reported using a fixed resolution of 448 × 448 pixels (16) or 1,024 × 1,024 pixels (17). Choosing the fixed resolution is a trade-off be computational efficiency and predictive accuracy (18). Therefore, storing the resized or down-sampled images can document the input into the CNN in an auditable and reproducible format.

**Figure 1:**
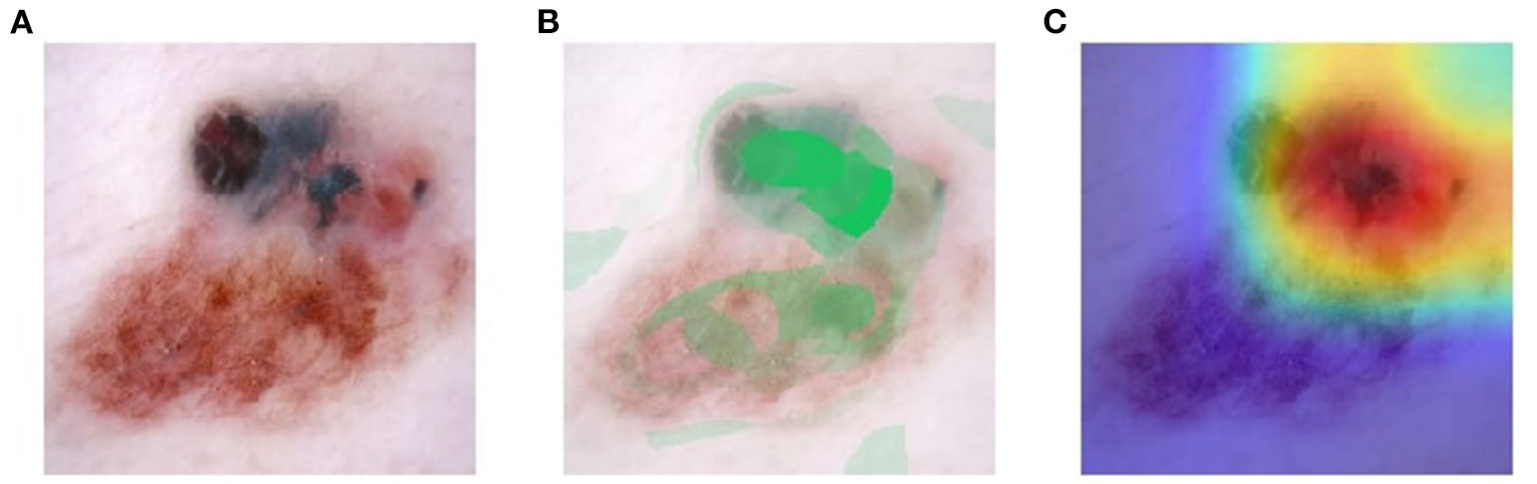
Visual explanation images where AI algorithm correctly classified the skin lesion as melanoma **(A)** original image **(B)** SHAP visual explanation where green indicates the most important area of contribution to the AI result and red indicates the least important area; and **(C)** Grad-CAM visual explanation image where red is the most important area of contribution to AI result and blue is the least important part of the image to AI result. Image courtesy of Sally Shrapnel.

**Figure 2:**
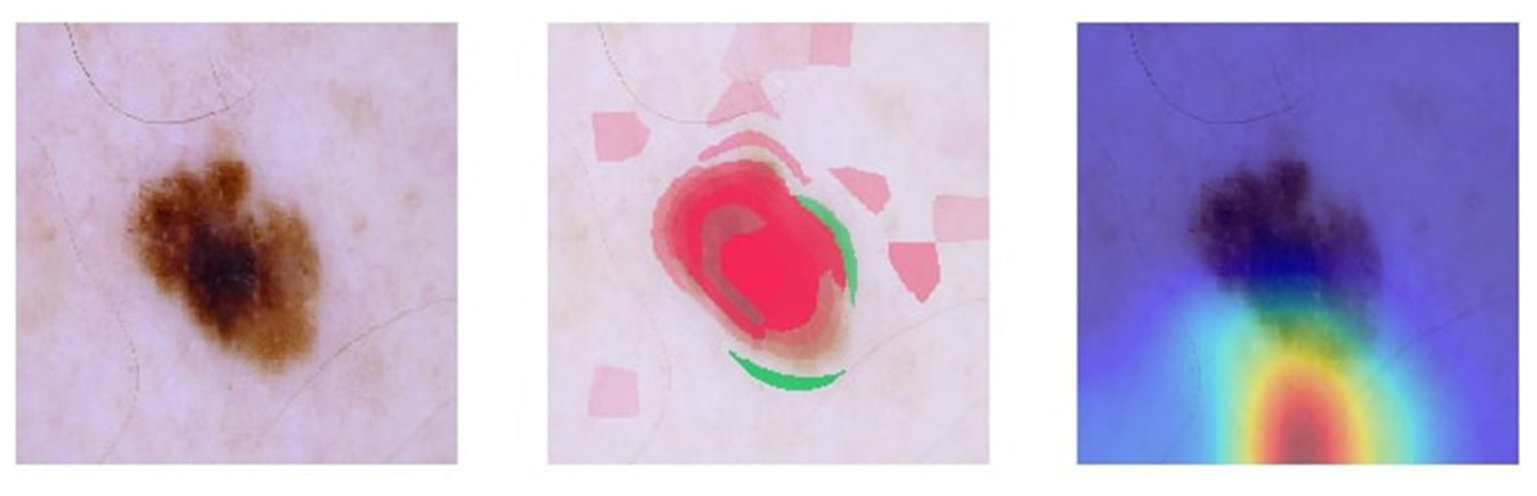
Visual explanation images where the AI algorithm incorrectly classified the skin lesion as benign. Note the pigmented lesion was not considered an important feature by either SHAP or Grad-CAM. Image courtesy of Sally Shrapnel.

Visual explanation maps assign each feature in an image a level of importance that the feature contributes to AI algorithm output. An example of two common visual explanation methods is Shapley additive explanation (SHAP) (19), and Gradient-Weighted Class Activation Mapping (Grad-CAM) (20). Examples of these visual explanations are shown in Figures 1, 2. Visual explanation maps can be used by clinicians to help them understand how the AI algorithm came to its output and to assess the quality of the output (21). Therefore, visual explanation maps can provide insight into potential sources of bias in AI algorithms (22). For example, if an AI algorithm predicted a high probability of melanoma but the visual explanation maps showed that the area of pigmentation in the lesion was not of high importance in determining this, then the clinician may be suspicious of the accuracy of the prediction (see Figure 2).

**Figure 3:**
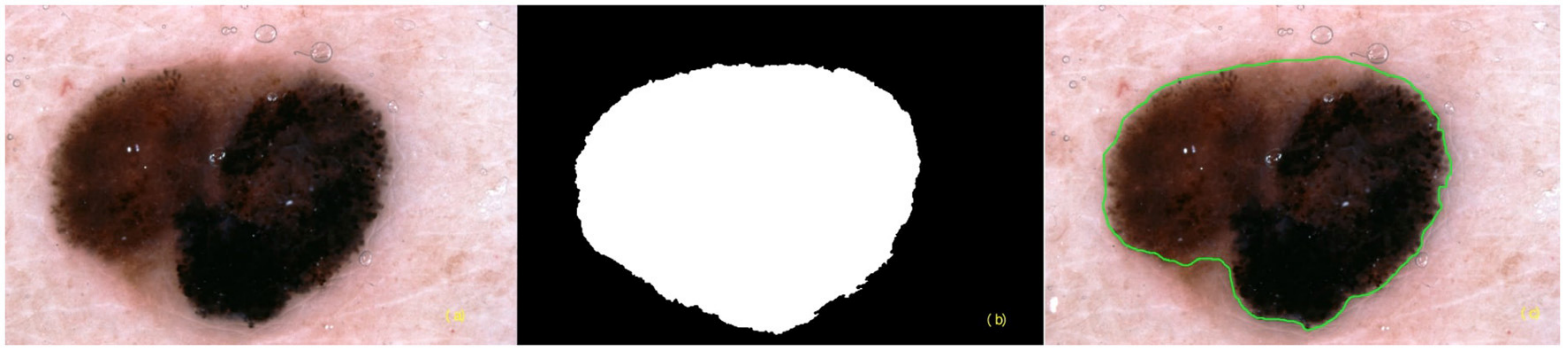
Segmentation images **(a)** referenced instance **(b)** binary segmentation instance **(c)** fractional segmentation instance.

Resized or down-sampled images can be encoded as DICOM photographic or secondary capture objects. DICOM segmentation objects can be used to encode both binary and fractional segmentation images as multi-frame images. The multiple frames include the referenced image instance and segmentation image instances (Figure 3). Visual explanation images can be encoded as parametric map objects. A current proposal is to be able to link segmentation objects and parametric map objects to DICOM Structured Reports (SR) for the measurement objects (23). DICOM is also trialing the use of Javascript Object Notation (JSON) for encoding the output of AI algorithms (e.g., risk prediction of skin disease) in DICOM SR format (24). The goal of this trial is to harmonize with the machine learning community where JSON is the preferred format for algorithm output. Additionally, JSON encoding will achieve encoding of ground truth diagnosis that can be used to train, test, and validate future machine learning algorithms.

## Metadata

The current use of CNNs for image classification has relied on image data as the only input. However, clinicians also rely on metadata as part of the diagnostic process (4). To emulate this, there have been several studies that have assessed the inclusion of metadata along with images as input into machine learning algorithms. One study found metadata resulted in a small improvement (1–2%) in sensitivity. Improvements were most noticeable in smaller, lower-performing models (25), which may indicate that the metadata compensates for fewer pixel data. A further study found the sensitivity and specificity improved from 82.1 to 95.2% and 86.1 to 91.0%, respectively (26). Both of these studies relied on simple metadata (age, sex, and lesion site). When lesion characteristics of bleeding, pain, and itchiness were added to age and the anatomical site as input, the accuracy of the algorithm improved by around 7% (27).

As previously noted, the DICOM file format (28) is an amalgamation of metadata with the pixel data of the image. The metadata is stored in the DICOM file header. The standardized metadata includes identifying and descriptive patient metadata (e.g., age, sex) and study metadata (e.g., lesion site). Most DICOM viewers allow the metadata encoded in the DICOM file header to be viewed in text format.

The DICOM supplement for dermoscopy has proposed the use of a skin cancer acquisition context. An acquisition context is a method of embedding descriptors of clinical metadata in the DICOM header using codes from standard lexicons [such as SNOMED CT (29)] for both concepts and values (30). The proposed skin cancer acquisition context includes rich clinical metadata such as a personal and familial history of melanoma, *in situ* melanoma, and other skin cancers; Fitzpatrick skin type; the degree of ultraviolet damage; atypical mole syndrome; and other relevant genetic conditions. Furthermore, the skin cancer acquisition context also includes lesion-level metadata such as history of growth, itch, erythema, and other relevant patient-observed changes. As DICOM is extensible, there is the ability to define acquisition contexts for different skin disease classes (e.g., inflammatory disease). There is the potential that using rich metadata stored in the acquisition context as an AI algorithm input will improve the performance of machine learning algorithms, perhaps more so than simple metadata. However, at this time it remains untested. The use of metadata is an area for further research, as it has been shown that not all metadata improve the performance of skin lesion image classifiers, and that selective metadata selection will likely be necessary (31).

## Datasets

One of the main limitations of deep learning is the input data used to train the system (32). AI models trained and validated from the same dataset risk overfitting, which is a phenomenon of “knowing the training data too well (33).” Overfitting results in the predictive output of an AI model only being reliable for the population on which the AI model was trained. To overcome this limitation, external validation or cross-validation is necessary and can be achieved by training the AI model on datasets amalgamated from multiple clinical sources, including different populations and ethnicities. Having images from these disparate sources in a standardized format facilitates the amalgamation of datasets and reduces the cost and effort of dataset curation. The lack of deployment of imaging and metadata standards in dermatology has inhibited the development of large image collections suitable for machine learning (34). DICOM is a universally recognized standard for medical imaging and would be ideal as a standardized image encoding to address this challenge.

The use of metadata-based retrieval (30), using queries based on DICOM metadata attributes, could also facilitate the creation of machine learning datasets with high levels of granularity. For example, to develop an AI algorithm specific to polarized light dermoscopy, one could assemble an appropriate dataset using query (based on DICOM attributes for the dermoscopy image type and polarization) and retrieve images from the repository where they are stored.

The amalgamation of datasets does increase the risk of unauthorized access to patient-identifiable information. DICOM's de-identification profiles (35), which are used to address privacy issues for multi-center clinical trials, can be directly applied to the amalgamation of machine learning datasets. The DICOM Attribute Confidentiality Profiles contain a comprehensive list of attributes that potentially provide identifying information and should be removed to protect the patient's privacy. Adopting an established de-identification profile has many advantages, including avoidance of repeating the same complex technical and legal consideration for de-identification, being less error-prone for participating sites to deploy, and implying a level of rigor that would potentially satisfy ethics committees.

Excluding images from datasets where the DICOM attribute Recognizable Visual Features (0028, 0302) is set is a further way to enhance the privacy of a dataset for machine learning. The Recognizable Visual Features flag is most often set when the subject's face is included in an image. In the forthcoming DICOM supplement for dermoscopy, this attribute is set if the images contain the patient's fingerprints.

## Discussion and Conclusion

The increasing use of imaging in dermatology is being driven by many factors, including diagnostic imaging (as opposed to documentary imaging) such as teledermatology, sequential dermoscopic imaging, and melanoma screening using total body photography (36). The ability of DICOM to encode relevant metadata and afford interoperability with the digital health ecosystem has driven the initial interest in the adoption of DICOM for dermatology (2). DICOM can improve AI workflows by encoding derived objects (e.g., secondary images, visual explainability maps, AI algorithm output) and the efficient curation of multi-institutional datasets for machine learning training, testing, and validation. This can be achieved using DICOM mechanisms such as standardized image formats and metadata, metadata-based image retrieval, and de-identification protocols.

Currently, the use of DICOM to manage dermatological imaging is not widespread. Further, the use of DICOM to support AI workflows in dermatology is likely more limited still. There has, however, been a successful proof-of-concept trial using DICOM to encode datasets for AI of skin disease (37). DICOM adoption by dermatology may be more challenging than for other medical specialties. Clinical photographs captured on a mobile device constitute most imaging in dermatology. This type of imaging lacks standardization (38). Cameras used for clinical photography in dermatology are often commercial-off-the-shelf products as opposed to dedicated medical devices, hence, unlikely to support DICOM. As opposed to other medical specialties where formal diagnostic reports are produced, dermatology images can be simply viewed, which lessens the importance of integration with PACS. There is a lot of enthusiasm for AI in dermatology (32). More widespread understanding of the role that DICOM has in facilitating the adoption of AI in dermatology may strongly influence the dermatology community to adopt DICOM.

AI workflows for dermatology would require some interoperable actors on a medical imaging network. A potential workflow would be that images are acquired and stored in an image archive using typical DICOM image acquisition and storage workflows. Images could be automatically routed from the archive to an AI evidence creator actor (e.g., CNN). The evidence creator may undertake image post-processing to produce resized or down-sampled images and/or segmentation objects. These images would be encoded as DICOM objects and be automatically stored in the image archive. The AI evidence creator would analyze the image of the skin and create evidence documents (e.g., DICOM SR) containing the risk prediction of skin disease and potentially visual explainability maps. Again, these derived objects would be encoded as DICOM objects and automatically stored in the image archive using DICOM network services. All derived objects created by the AI evidence creator would use the same study identifier as the original images to facilitate linkage. A dermatologist or other clinician could use a DICOM viewer to subsequently query and retrieve an imaging study and display the original images and derived objects (e.g., a structured report containing algorithm output and/or visual explainability maps) that would help with image interpretation. DICOM-facilitated interoperability between AI actors and other devices in a medical imaging network is likely to facilitate workflow efficiencies. In addition to the transmission of images and results, interoperability is likely to play a key role in the continual adaptive learning of machine learning algorithms (39). Interoperability will also allow health-care organizations to use “best-of-breed” AI actors rather than being locked into an existing vendor's product.

The use of DICOM for the management of dermatological images will not guarantee effective clinical use of AI in dermatology. DICOM can address several important technological and workflow challenges for the implementation of AI. However, many other technological, ethical, regulatory, medicolegal, and workforce barriers will need to be addressed before DICOM and AI can be used effectively in dermatology.

## Data Availability Statement

The raw data supporting the conclusions of this article will be made available by the authors, without undue reservation.

## Author Contributions

All authors contributed equally to the preparation of this perspective piece.

## Conflict of Interest

HS is a shareholder of MoleMap NZ Limited and e-derm consult GmbH, and undertakes regular teledermatological reporting for both companies. HS also provides medical consultant services for Canfield Scientific Inc., First Derm by iDoc24 Inc, and Revenio Research Oy. DC provides consultancy to: MITA (editor of DICOM standard), Canfield Scientific, Philips Algotec, Essex Leidos CBIIT NCI, Brigham and Women's Hospital NCI Imaging Data Commons (IDC), University of Leeds Northern Pathology Imaging Co-operative (NPIC) and is on the advisory board of maiData. The remaining authors declare that the research was conducted in the absence of any commercial or financial relationships that could be construed as a potential conflict of interest.

## References

[1] National Electrical Manufacturers Association About DICOM: Overview. National Electrical Manufacturers Association (2020). Available online at: https://www.dicomstandard.org/current (accessed September 01, 2020).

[2] Caffery LJ Clunie D Curiel-Lewandrowski C Malvehy J Soyer HP Halpern AC. Transforming dermatologic imaging for the digital era: metadata and standards. J Digit Imaging. (2018) 31:568–77. 10.1007/s10278-017-0045-8 29344752 PMC6113154

[3] DICOM Standards Committee Supplement 221: Dermoscopy (Letter Ballot). (2020). Available online at: ftp://medical.nema.org/MEDICAL/Dicom/Supps/LB/sup221_lb_dermoscopy.pdf (accessed October 01, 2020).

[4] Wada M Ge Z Gilmore SJ Mar VJ. Use of artificial intelligence in skin cancer diagnosis and management. Med J Aust. (2020) 213:256–9.e1. 10.5694/mja2.50759 32892366

[5] Esteva A Kuprel B Novoa RA Ko J Swetter SM Blau HM. Dermatologist-level classification of skin cancer with deep neural networks. Nature. (2017) 542:115–8. 10.1038/nature21056 28117445 PMC8382232

[6] Haenssle HA Fink C Schneiderbauer R Toberer F Buhl T Blum A. Man against machine: diagnostic performance of a deep learning convolutional neural network for dermoscopic melanoma recognition in comparison to 58 dermatologists. Ann Oncol. (2018) 29:1836–42. 10.1093/annonc/mdy166 29846502

[7] Tschandl P Rinner C Apalla Z Argenziano G Codella N Halpern A. Human-computer collaboration for skin cancer recognition. Nat Med. (2020) 26:1229–34. 10.1038/s41591-020-0942-0 32572267

[8] Zhao S Xie B Li Y Zhao X Kuang Y Su J. Smart identification of psoriasis by images using convolutional neural networks: a case study in China. J Eur Acad Dermatol Venereol. (2020) 34:518–24. 10.1111/jdv.15965 31541556

[9] Han SS Park GH Lim W Kim MS Na JI Park I. Deep neural networks show an equivalent and often superior performance to dermatologists in onychomycosis diagnosis: automatic construction of onychomycosis datasets by region-based convolutional deep neural network. PLoS ONE. (2018) 13:e0191493. 10.1371/journal.pone.0191493 29352285 PMC5774804

[10] National Electrical Manufactuerres Association AI and DICOM. (2020). Available online at: https://www.dicomstandard.org/ai (accessed October 01, 2020).

[11] Haenssle HA Fink C Toberer F Winkler J Stolz W Deinlein T. Man against machine reloaded: performance of a market-approved convolutional neural network in classifying a broad spectrum of skin lesions in comparison with 96 dermatologists working under less artificial conditions. Ann Oncol. (2020) 31:137–43. 10.1016/j.annonc.2019.10.013 31912788

[12] Geis JR Brady AP Wu CC Spencer J Ranschaert E Jaremko JL. Ethics of artificial intelligence in radiology: summary of the joint European and North American multisociety statement. Radiology. (2019) 293:436–40. 10.1148/radiol.2019191586 31573399

[13] Booz C Yel I Wichmann JL Boettger S Al Kamali A Albrecht MH. Artificial intelligence in bone age assessment: accuracy and efficiency of a novel fully automated algorithm compared to the greulich-pyle method. Eur Radiol Exp. (2020) 4:6. 10.1186/s41747-019-0139-9 31993795 PMC6987270

[14] Conant EF Toledano AY Periaswamy S Fotin SV Go J Boatsman JE. Improving accuracy and efficiency with concurrent use of artificial intelligence for digital breast tomosynthesis. Radiol Artif Intell. (2019) 1:e180096. 10.1148/ryai.2019180096 32076660 PMC6677281

[15] Pesapane F Codari M Sardanelli F. Artificial intelligence in medical imaging: threat or opportunity? radiologists again at the forefront of innovation in medicine. Eur Radiol Exp. (2018) 2:35. 10.1186/s41747-018-0061-6 30353365 PMC6199205

[16] Mahbod A Tschandl P Langs G Ecker R Ellinger I. The effects of skin lesion segmentation on the performance of dermatoscopic image classification. Comput Methods Programs Biomed. (2020) 197:105725. 10.1016/j.cmpb.2020.105725 32882594

[17] Combalia M Codella NC Rotemberg V Helba B Vilaplana V Reiter O BCN20000: dermoscopic lesions in the wild. arXiv. (2019). *arXiv* 1908.02288v2. 10.1038/s41597-024-03387-w PMC11183228 38886204

[18] Hashemi M Enlarging smaller images before inputting into convolutional neural network: zero-padding vs. interpolation. J Big Data. (2019) 6:98 10.1186/s40537-019-0263-7

[19] Lundberg SM Lee S-I A unified approach to interpreting model predictions. In: von Luxburg U Guyon I, editors. Proceedings of the 31st International Conference on Neural Information Processing Systems. Long Beach, CA: Curran Associates Inc, (2017). p. 4765–74.

[20] Selvaraju RR Cogswell M Das A Vedantam R Parikh D Batra D Grad-CAM: visual explanations from deep networks via gradient-based localization. Int J Comput Vision. (2020) 128:336–59. 10.1007/s11263-019-01228-7

[21] Holzinger A Langs G Denk H Zatloukal K Muller H. Causability and explainability of artificial intelligence in medicine. Wiley Interdiscip Rev Data Min Knowl Discov. (2019) 9:e1312. 10.1002/widm.1312 32089788 PMC7017860

[22] Young K Booth G Simpson B Dutton R Shrapnel S Deep neural network or dermatologist? In: Suzuki K Reyes M Syeda-Mahmood T editors. Interpretability of Machine Intelligence in Medical Image Computing and Multimodal Learning for Clinical Decision Support: Proceedings of the Second International Workshop on Interpretability of Machine Intelligence in Medical Image Computing, iMIMIC 2019, and the 9th International Workshop on Multimodal Learning for Clinical Decision Support, ML-CDS 2019, held in conjunction with the 22nd International Conference on Medical Imaging and Computer-Assisted Intervention, MICCAI 2019. Shenzhen: Springer International Publishing (2019). p. 48–55.

[23] DICOM Standards Committee CP-1867 Add codes for visual Explanation maps. (2020). Available online at: https://dicom.nema.org/Dicom/News/September2019/docs/cpack103/cp1867.pdf (accessed October 01, 2020).

[24] DICOM Standards Commiteee Supplement 219: JSON Representation of DICOM Structured Reports. (2020). Available online at: ftp://medical.nema.org/medical/dicom/supps/Frozen/sup219_fz_14_JSONSR.pdf (accessed October 01, 2020)

[25] Gessert N Nielsen M Shaikh M Werner R Schlaefer A. Skin lesion classification using ensembles of multi-resolution efficientnets with meta data. MethodsX. (2020) 7:100864. 10.1016/j.mex.2020.100864 32292713 PMC7150512

[26] Liu Z Sun J Smith M Smith L Warr R. Incorporating clinical metadata with digital image features for automated identification of cutaneous melanoma. Br J Dermatol. (2013) 169:1034–40. 10.1111/bjd.12550 23902335

[27] Pacheco AGC Krohling RA. The impact of patient clinical information on automated skin cancer detection. Comput Biol Med. (2020) 116:103545. 10.1016/j.compbiomed.2019.103545 31760271

[28] National Electrical Manufacturers Association Digital Imaging and Communications in Medicine (DICOM) Standard PS3.10 - Media Storage and File Format for Media Interchange. (2020). Available online at: https://dicom.nema.org/medical/dicom/current/output/pdf/part10.pdf (accessed October 01, 2020).

[29] Donnelly K. SNOMED-CT: the advanced terminology and coding system for eHealth. Stud Health Technol Inform. (2006) 121:279–90. 17095826

[30] Bidgood WD Jr Bray B Brown N Mori AR Spackman KA Golichowski A. Image acquisition context: procedure description attributes for clinically relevant indexing and selective retrieval of biomedical images. J Am Med Inform Assoc. (1999) 6:61–75. 10.1136/jamia.1999.0060061 9925229 PMC61345

[31] Li W Zhuang J Wang R Zhang J Zheng W Fusing metadata and dermoscopy images for skin disease diagnosis. In: 2020 IEEE 17th International Symposium on Biomedical Imaging (ISBI). (2020). p. 1996–2000.

[32] Du-Harpur X Watt FM Luscombe NM Lynch MD. What is AI? Applications of artificial intelligence to dermatology. Br J Dermatol. (2020) 183:423–30. 10.1111/bjd.18880 31960407 PMC7497072

[33] Chan S Reddy V Myers B Thibodeaux Q Brownstone N Liao W. Machine learning in dermatology: current applications, opportunities, and limitations. Dermatol Ther. (2020) 10:365–86. 10.1007/s13555-020-00372-0 32253623 PMC7211783

[34] Curiel-Lewandrowski C Novoa RA Berry E Celebi ME Codella N Giuste F Artificial Intelligence Approach in Melanoma. Melanoma. New York, NY: Springer (2019). p. 1–31.

[35] National Electrical Manufacturers Association Digital Imaging and Communications in Medicine (DICOM) Standard PS3.15 - Security and System Management Profiles - E.1 Attribute Confidentiality Profiles - De-identifier. (2020). Available online at: https://dicom.nema.org/medical/dicom/current/output/chtml/part15/chapter_E.html#sect_E.1.1 (accessed October 01, 2020)

[36] Rayner JE Laino AM Nufer KL Adams L Raphael AP Menzies SW. Clinical perspective of 3D total body photography for early detection and screening of Melanoma. Front Med. (2018) 5:152. 10.3389/fmed.2018.00152 29911103 PMC5992425

[37] Rotemberg V Kurtansky N Betz-Stablein B Caffery L Chousakos E Codella N. A patient-centric dataset of images and metadata for identifying melanomas using clinical context. arXiv. (2020). *arXiv* 2008.07360v1. 33510154 10.1038/s41597-021-00815-z PMC7843971

[38] Quigley EA Tokay BA Jewell ST Marchetti MA Halpern AC. Technology and technique standards for camera-acquired digital dermatologic images: a systematic review. JAMA Dermatol. (2015) 151:883–90. 10.1001/jamadermatol.2015.33 25970844

[39] Dikici E Bigelow M Prevedello L White R Erdal B. Integrating AI into radiology workflow: levels of research, production, and feedback maturity. J Med Imaging. (2020) 7:016502. 10.1117/1.JMI.7.1.016502 32064302 PMC7012173

